# Adaptation and initial psychometric study of the anxiety and fear of COVID-19 scale in the United Kingdom population

**DOI:** 10.3389/fpsyt.2023.1071146

**Published:** 2023-02-06

**Authors:** Cristina Morgado-Toscano, Regina Allande-Cussó, Javier Fagundo-Rivera, Yolanda Navarro-Abal, Jose Antonio Climent-Rodríguez, Juan Gómez-Salgado

**Affiliations:** ^1^Health Sciences Doctorate, University of Huelva, Huelva, Spain; ^2^Health Sciences Research Unit: Nursing, University of Coimbra, Coimbra, Portugal; ^3^Nursing, University of Seville, Seville, Spain; ^4^Centro Universitario de Enfermería Cruz Roja, University of Seville, Seville, Spain; ^5^Faculty of Labour Sciences, University of Huelva, Huelva, Spain; ^6^Department of Sociology, Social Work and Public Health, Faculty of Labour Sciences, University of Huelva, Huelva, Spain; ^7^Safety and Health Postgraduate Programme, Escuela de Posgrado, Universidad de Especialidades Espíritu Santo, Guayaquil, Ecuador

**Keywords:** anxiety, COVID-19, fear, scales, United Kingdom

## Abstract

**Objective:**

The aim of the study was the initial psychometric study to validate the anxiety and fear of COVID-19 (AMICO) assessment scale in the general population of the United Kingdom population.

**Materials and methods:**

A descriptive, cross-sectional, psychometric validation and descriptive study was conducted, performing univariate and bivariate analyses, as well as exploratory and confirmatory factor analysis.

**Results:**

The sample was 658 people living in the United Kingdom over 16 years. Of the total, 80.5% were female, with a mean age of 48.25 years (SD = 14.861). A mean score for the AMICO scale of 4.85 (SD = 2.398) was obtained, with a range of scores from 1 to 10. The study of percentiles and quartiles allowed for the identification of three proposed levels of anxiety.

**Conclusion:**

The AMICO_UK scale is reliable to measure the presence of anxiety and fear related to the COVID-19 disease in the United Kingdom population. The majority of the United Kingdom population presented low levels of anxiety and fear at the time the scale was administered.

## Highlights

– One of the main consequences of the pandemic in the United Kingdom has been an increase in social isolation and feelings of loneliness.
– this pandemic context, there is a need to create tools to measure the impact on the mental health of people.
– The AMICO_UK scale shows statistically significant differences with the variables sex, income level at the end of the month, health status score, self- confinement, amount of information received, and vaccine side effects.
– The results have provided optimal outcomes in the goodness-of-fit indices for the construct validation of the AMICO_UK scale and its overall reliability.

## 1. Introduction

In December 2019, a new form of pneumonia of unknown origin was identified in Wuhan, China. On January 12, 2020, Chinese authorities revealed the sequence of a new, hitherto unknown virus called SARS-CoV-2 as the cause of this pneumonia. Shortly afterwards, at the end of February 2020, the virus had already spread rapidly in China, but also in 28 other countries. The pandemic was thus declared on March 11, 2020, when the virus was already circulating on all 5 continents (1).

The United Kingdom was one of the first countries affected in Europe, with the first confirmed cases of COVID-19 detected on January 31, 2020 (2). Following detection, Public Health England, the national public health agency, developed a surveillance system called First Few X for COVID-19 (2). Currently, an estimated 19,820,181 cases have been reported in the United Kingdom since the start of the pandemic and up to 163,095 deaths (3).

The most frequently reported symptoms were fever, fatigue, dry cough, myalgia, and dyspnoea (4). In addition to this, studies have included a worsening of the population’s mental health and increased risk of psychiatric illness in the aftermath of the COVID-19 pandemic (5).

According to various theories such as the one on stress and the one that focuses on perceived risk, negative emotions develop in public emergencies. If these negative emotions are sustained over time, they can even affect the immune function of the population, making them more vulnerable to suffer from the disease which, in this case, is caused by the new virus (6). Therefore, in addition to the organic and physiological effects, the consequences caused by COVID-19 on the physical and mental health of the population have even been defined as ‘coronaphobia’ (7).

The impact that COVID-19 may have on mental health is still being evaluated worldwide, but the associated interventions and services remain largely unstudied. COVID-19 clearly is a health threat identified as a significant stressor (8). Stressful life events, such as those triggered by the COVID-19 pandemic, have a significant influence on an individual’s psychological functioning and well-being, and can act as catalysts for psychological problems including anxiety, confusion, social isolation, and depression (9). Individual differences regarding resilience, coping, and perceptions can influence how a person responds to adverse experiences.

At the start of the first confinement in the United Kingdom on March 23, 2020, rates of psychological distress were found to be significantly higher than in the 6 years prior to the arrival of COVID-19 (10). Another study noted that the rate of psychological distress in the United Kingdom adult population in 2020 was much higher than data collected in 2018, with 18–24 year olds and women being most affected (11). Likewise, one of the main consequences of the pandemic has been an increase in social isolation and feelings of loneliness. This is highly correlated with symptoms of anxiety, depression, and suicidal ideation (12). Fear, understood as a cognitive response to a threat (13), favours human adaptation to certain dangers, but if it remains over time or becomes characteristic of the individual, it can predispose to the appearance of physical illnesses and/or psychological disorders, or aggravate previous pathologies (14). In addition to psychological consequences, long-term mental illnesses and social tension predominate (15).

In pandemic times, people may experience a wide range of psychological states such as fear and anxiety, which may be exacerbated by measures of imposed social isolation and feelings of loneliness (15, 16). Anxiety, which is defined as feelings of tension, worrying thoughts, and physical changes in the body, is the most common psychological problem experienced during a pandemic. In the case of fear, it is defined as a negative emotion accompanied by a high level of nervousness and is evoked by a threat that is considered to have significant consequences for a person (16). When people face challenges during traumatic experiences, their self-esteem, trust in others, self-control, and predictability of the world may be damaged, and this can lead to unpleasant attitudes towards themselves and others. People who experience a high level of fear tend to believe that negative things can happen to them again when confronted with traumatic events (17).

In this pandemic context, it is necessary to create tools that specifically measure the impact on the mental health of people to be able to design future interventions according to population needs. One of the first scales designed specifically for this purpose was the fear of COVID-19 (FCV-19), created by Ahorsu et al. This scale consists of 10 items and has shown to have good psychometric properties in the Iranian population, being subsequently validated in several countries (18). On the other hand, Silva et al. validated the COVID anxiety scale (CAS-19), with 7 items and optimal fit values, to assess the presence of COVID-19 anxiety in the Brazilian population (19).

In the case of Spain, a group of researchers designed and validated a scale that measured not only the fear dimension of COVID-19, but also anxiety. This scale, which was named AMICO (for its Spanish acronym); Escala de Evaluación de la Ansiedad y MIedo a COVID-19, or Scale for the Evaluation of Anxiety and Fear of COVID-19, evaluates the anxiety and fear constructs in a single measurement scale, given the moderate correlation between the two constructs, although different, demonstrated in the literature (20). The questionnaire consists of 16 items, with two factors (anxiety and fear), and proved to be reliable and valid to specifically measure fear and anxiety related to COVID-19 (21).

In the specific case of the United Kingdom, different studies have assessed the presence of COVID-19 anxiety in its population since the beginning of the pandemic, but never used a measurement scale that brought together the constructs of anxiety and fear (9, 17–19). In this context, the present study aims to cross-culturally validate and to conduct the initial psychometric study of the AMICO scale in the United Kingdom, as a screening tool for anxiety and fear of COVID-19 in this population, carrying out the corresponding cross-cultural adaptation.

## 2. Methods

### 2.1. Design

Descriptive, cross-sectional study of psychometric validation and cross-cultural adaptation of a previously validated questionnaire.

### 2.2. Instrument

The initial scale used for this study was the anxiety and fear of COVID-19 assessment scale (AMICO). After the process of creating and designing the scale, it proved to be a reliable and valid tool to be used as a screening instrument for the presence of anxiety and fear in the Spanish population (22).

### 2.3. Participants and procedures

This study was conducted in the United Kingdom, a country with a population of 67,025,542 as of 2019 (23). A sample of at least 239 people was estimated for this study, with a confidence level of 95%, a precision of 3%, and an expected loss ratio of 15%. Finally, the total sample for this study was 658. For the adaptation of the AMICO scale to the United Kingdom context, a process of direct translation into English and back-translation back into Spanish was first carried out to verify that the translated version reflected the same content as the original versions (24).

This double translation was carried out by two native Spanish translators, with experience in translating scientific documents, and who lived and worked in the United Kingdom. Their minimum level of education was a Master’s degree. The English-translated version was evaluated by a panel of 10 experts to assess its cultural adaptation to the United Kingdom context and to consolidate the versions of the questionnaire (25). These experts, who were working in the National Health Service and with a minimum level of a Master’s degree, were identified and contacted by the authors of this study. The version adapted to the United Kingdom context was given the name “AMICO_UK.”

Once the AMICO_UK scale was agreed by the panel of experts, a pilot test was carried out with 20 subjects to detect comprehension problems. An online questionnaire was set up using the GoogleForms© application with the final version of the scale. After piloting, the field study began with a sample of subjects residing in the United Kingdom and aged 16 and over. A non-probabilistic snowball sampling was carried out during the months of April and June 2021, through different social networks, sending them the link to access the survey. In order to participate in the study, it was necessary to give voluntary consent.

Once the options of voluntary participation and informed consent had been selected, subjects could access the survey. In addition to this, they also had access to an introductory text informing them about the study, as well as about the research team and their contact details.

### 2.4. Variables

The questionnaire, in addition to the AMICO_UK scale, contained demographic variables (age, sex, city, marriage, educational level, cohabitant, and household size). Employment variables were also included, such as employment situation, area of work and salary, as well as health variables such as various questions related to COVID-19, COVID-19 vaccination status, and possible side effects of COVID-19. It should be noted that the sample consisted of people who were currently residing in the United Kingdom, although neither the nationality variable nor the length of time they had been residing in the United Kingdom was recorded (26).

### 2.5. Data analysis

Univariate and bivariate descriptive analysis were calculated with the SPSS Statistics © v26 (27) software. The Kolmogorov–Smirnov test was performed, obtaining a value of *p* < 0.005, so the data distribution was considered non-normal and non-parametric tests were used in the analysis. The Mann–Whitney U and Kruskal–Wallis tests were used for contrast tests. Kendall’s Tau-b test was also used to study the correlation between two quantitative variables.

To establish the relationship between the presence of anxiety and fear (measured with the AMICO questionnaire) and psychological distress (assessed with the GHQ-12 questionnaire) with the rest of the independent variables within each subsample, categorical regression analysis (CATREG) was performed, according to the qualitative nature of these variables (28).

To investigate the factor structure of the AMICO_UK scale, an exploratory factor analysis was performed, using principal axial factoring, as it is suitable when the distribution of the data does not follow normality and stable factorial solutions are sought, and promax rotation, which allows the factors to be correlated (29). For the configuration of the final factorial solution, the highest weights for each item were selected in both factors. Finally, the Kaiser-Guttman criterion was applied to define the number of factors, considering eigenvalues greater than 0.961. In addition, to ensure that the final factor solution was not one factor, a confirmatory factor analysis was performed considering a single factor, following the recommendations of Podsakoff et al., based on Harman’s single factor test (30).

Regarding the confirmatory factor analysis, an unweighted least square (ULS) estimation procedure was used, as the observed indicators did not follow a continuous normal distribution. The next means were utilized to measure the goodness of fit of the confirmatory model: the Tucker-Lewis index (TLI) (values ≥0.96 suggesting a good fit); the penalty function Chi-squared degrees of freedom (CMIN/DF) (values ≤3 showed a good fit); the normalised fit index (NFI); the comparative fit index (CFI); the standardized root mean square residual (SRMR) (values ≤0.80 suggested a good fit), and the root mean square error of approximation (RMSEA) (values ≤0. 05 or 0.08 showed a good fit).

Subsequently, to study the unidimensionality of the scale, a bifactor model was analyzed, considering the same first-order factors validated by means of the recently carried out CFA and also a second-order factor (bifactor) where each item was also subsumed. For the calculation of these goodness-of-fit parameters of the bifactor model, Dueber’s bifactor index calculator (31) was used. Specifically, the percent of uncontaminated correlations (PUC) was used, which represents the percentage of variance that corresponds only to the overall dimension, the percentage of explained common variance (ECV), which is the proportion of total variance that is explained by each factor (general and specific) and the Omega Hierarchical (OmegaH), which reflects the percentage of systematic variance of the total score that can be attributed to individual differences in the general factor. Regarding the cut-off points for these indices, Reise et al. suggest that PUC values >80, together with LCS values >60 and Omega H >80, would indicate that the presence of multidimensionality would not be too severe to rule out unidimensionality of the scale (32).

For the reliability study, Cronbach’s alpha was calculated. Furthermore, based on new recommendations for the study on the reliability of the measurement scales, the McDonald’s omega coefficient was calculated, which confirms the premise of Tau equivalence and is a more robust indicator of the reliability of the scale (33). In addition, the McDonald’s omega coefficient was corrected, considering the impact of correlated errors on the reliability indices (34).

As no scale was used as a gold standard, it was not possible to run the ROC curve. However, three levels of anxiety and fear of COVID-19 were identified from the study of percentiles and the bivariate analysis between each of the levels.

### 2.6. Ethical considerations

Permission to conduct this study was obtained from the National Health Service (NHS) Committee, Ref. 20/HRA/369. This study also complies with the Declaration of Helsinki: Ethical Principles for Medical Research Involving Human Subjects guidelines (35) in its latest edition (Fortaleza, Brazil, 2013).

The subjects who made up the panel of experts were identified by their affiliation with the National Health Service through direct consultation on the agency’s official website. For this purpose, they were sent a participation email that contained, in addition to information on the project, the express request to participate as an expert under the premises of confidentiality and voluntariness.

In order to participate in the present study, both for the panel of experts and for the subjects of the scale validation study, it was necessary for the sample to confirm their voluntary and confidential participation in the study through a specific box in order to be able to access the questionnaire.

## 3. Results

### 3.1. Descriptive analysis

The sample that completed the online questionnaire consisted of 658 subjects, all over 16 years of age and residing in the United Kingdom. Of the total sample, 80.5% were female, with a mean age of 48.25 years (SD = 14.861).

In terms of income, 71.7% said they had sufficient resources to make ends meet. With regard to general health status, the mean score was 6.67. Also, 58.8% had never self- confined. In general, they considered themselves well informed about the pandemic. Only 14.5% of the sample had been vaccinated with both doses and 62.6% had received only one dose; of those who had, 35.6% had had no side effects after administration of the vaccine.

Regarding the AMICO scale variable, the mean obtained for the total score on the AMICO scale was 4.85 (SD = 2.398), with a range of scores from 1 to 10 (Table 1).

**Table 1:** Description of the sample profile.

Variables	Total sample (*n* = 658)	Mean AMICO_UK score (SD)	Contrast hypothesis
**Sex**
Male	129 (18.3%)	4.15 (2.20)	*p* = 0.000^a^
Female	529 (80.5%)	5.09 (2.41)	
**Enough income**
Yes	471 (71.7%)	4.61 (2.27)	
No	58 (8.8%)	5.94 (2.48)	
Not always	103 (15.7%)	5.64 (2.58)	*p* = 0.000^c^
Rather not say	20 (3%)	4.93 (2.84)	
Other	5 (0.8%)	5.03 (2.19)	
**Self-confinement**
No	264 (40.2%)	4.31 (2.42)	*p* = 0.000^a^
Yes	386 (58.8%)	5.76 (2.22)	
**Vaccination**
Yes, 2 doses	95 (14.5%)	4.60 (2.22)	
Yes, 1 dose	411 (62.6%)	5.16 (2.40)	
No	105 (16%)	5.11 (2.22)	P = 0.000^c^
Does not want to get vaccinated	41 (6.2%)	2.53 (1.65)	
Rather not say	5 (0.8%)	4.00 (3.70)	
**Side effects**
I have not been vaccinated	142 (21.6%)	4.35 (2.40)	
No side effects, except for little pain in the injection site	234 (35.6%)	4.69 (2.31)	
Rather not say	7 (1.1%)	3.79 (1.50)	
Yes, after the first and second doses	78 (11.9%)	5.95 (2.22)	*p* = 0.000^c^
Yes, only after the first dose	171 (26%)	5.95 (2.2)	
Yes, only after the second dose	25 (3.8%)	5.22 (2.38)	
**Age**
Mean (SD)	48.25 (14.86)		Tau b = 0.047^b^
**Health state score**
Mean (SD)	6.67 (2.06)		Tau b = 0.2757^b^
**Amount of information**
Mean (SD)	7.94 (2.07)		Tau b = 0.120^b^

^a^U Mann–Whitney; ^b^Kendall Tau-B; ^c^ANOVA.

On the other hand, the Kolmogorov–Smirnov test, with a significance of 0.000, revealed that the scores obtained on the AMICO_UK scale did not follow a normal distribution. The bivariate analysis revealed statistically significant differences in relation to the variables sex, income level at the end of the month, health status score, self-confinement, amount of information received, vaccination, and side effects of the vaccine in relation to the mean AMICO_UK scores (Table 1).

Furthermore, categorical regression analysis performed with the mean total score of the AMICO questionnaire, as the dependent variable and the remaining variables that showed significant differences in the bivariate analysis, revealed a R^2^ value of 0.48 and a value of *p* = 0.001 (see Table 2). Regression results indicated that men had 0.12 times less anxiety and fear of COVID-19 (coefficient *β* = −0.12; *F* = 9.56; *p* = 0.002); similarly, people with sufficient income had 0.16 times less anxiety and fear of COVID-19 (coefficient *β* = −0.169; *F* = 17.01; *p* = 0.001). Additionally, people who self-confirmed had 2.20 times more anxiety and fear of COVID-19 (coefficient *β* = 0.220; *F* = 17.96; *p* = 0.001), and people who had not been vaccinated or had only one dose had 0.25 times more anxiety than those who had been vaccinated (coefficient *β* = 0.256; *F* = 5.70; *p* = 0.01).

**Table 2:** Model adjustment and significance of the regression analysis.

*R*^2^ = 0.44	AMICO scale			
	Fisher’s *F* = 18.00		*p* = 0.001	
Variable	Coefficient	Degrees of freedom	Fisher’s F	*p*-value
Sex	−0.121	1	9.566	0.002
Enough income	−0.169	4	17.017	0.001
Self-confinement	0.220	2	17.960	0.001
Vaccination	0.256	1	5.706	0.017
Side effects	0.085	1	2.017	0.156

### 3.2. Psychometric analysis

Prior to the exploratory factor analysis (EFA), a Kaiser-Meyer-Olkin measure of 0.961 and a significance level of 0.000 in the Barlett’s test of sphericity were obtained. With these results, the EFA was implemented, under the criteria of principal axial factoring and promax rotation, which yielded a factorial solution of 2 dimensions and 16 items (Table 3). This factorial solution explained 68% of the variance.

**Table 3:** Rotated component matrix.

	Factor	
	Fear	Anxiety
AMICO_1	0.860	0.607
AMICO_2	0.877	0.608
AMICO_3	0.848	0.671
AMICO_4	0.836	0.600
AMICO_5	0.563	0.792
AMICO_6	0.179	0.798
AMICO_7	0.597	0.878
AMICO_8	0.644	0.890
AMICO_9	0.665	0.719
AMICO_10	0.364	0.712
AMICO_11	0.813	0.614
AMICO_12	0.603	0.495
AMICO_13	0.881	0.660
AMICO_14	0.844	0.624
AMICO_15	0.826	0.764
AMICO_16	0.803	0.705

**Figure 1:**
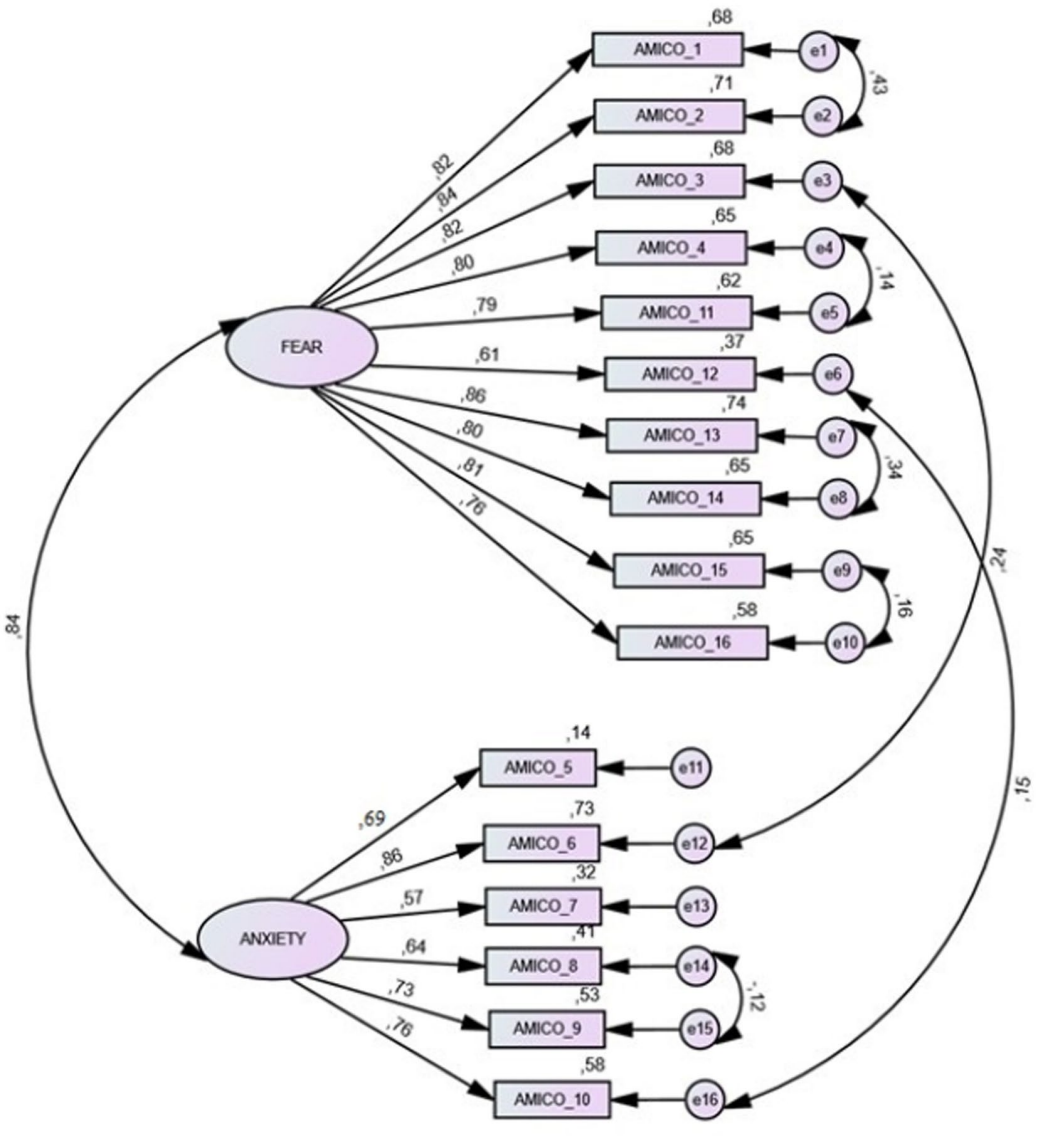
Confirmatory factor analysis.

A reliability study was also conducted, which produced an overall Cronbach’s α value of 0.964, 0.90, and 0.92 for each of the factors. The McDonald’s omega coefficient value for the composite reliability study was 0.92. The omega correction was run, taking into account the correlation between the errors, and a corrected omega value of 0.91 was obtained. Subsequently, a confirmatory factor analysis (CFA) was performed for the construct validity study, which gave the following values: CMIN/DF = 4.59 *p* = 0.17=; NFI = 0.93; TLI = 0.946; CFI = 0.946; RMSEA = 0.07; and SRMR = 0.04 (Figure 1). Although Kaiser’s rule showed the existence of two factors, another confirmatory factor analysis considering a single factor was implemented to assess the relevance of a bifactorial model. The results of these second CFA showed suboptimal fit values, compared to those obtained using the CFA with a two-factor model: CMIN/DF = 4.59 *p* = 0.17; NFI = 0.97; TLI = 0.80; CFI = 0.81; RMSEA = 0.15; and SRMR = 0.10.

In relation to the Bifactor parameters, values of PUC = 0.50, ECV = 0.68, and Omega H = 0.84 were obtained, suggesting that the presence of some multidimensionality is not severe enough to disqualify the interpretation of the instrument as primarily unidimensional. Therefore, the final score of only one on the AMICO scale can be considered.

**Figure 2:**
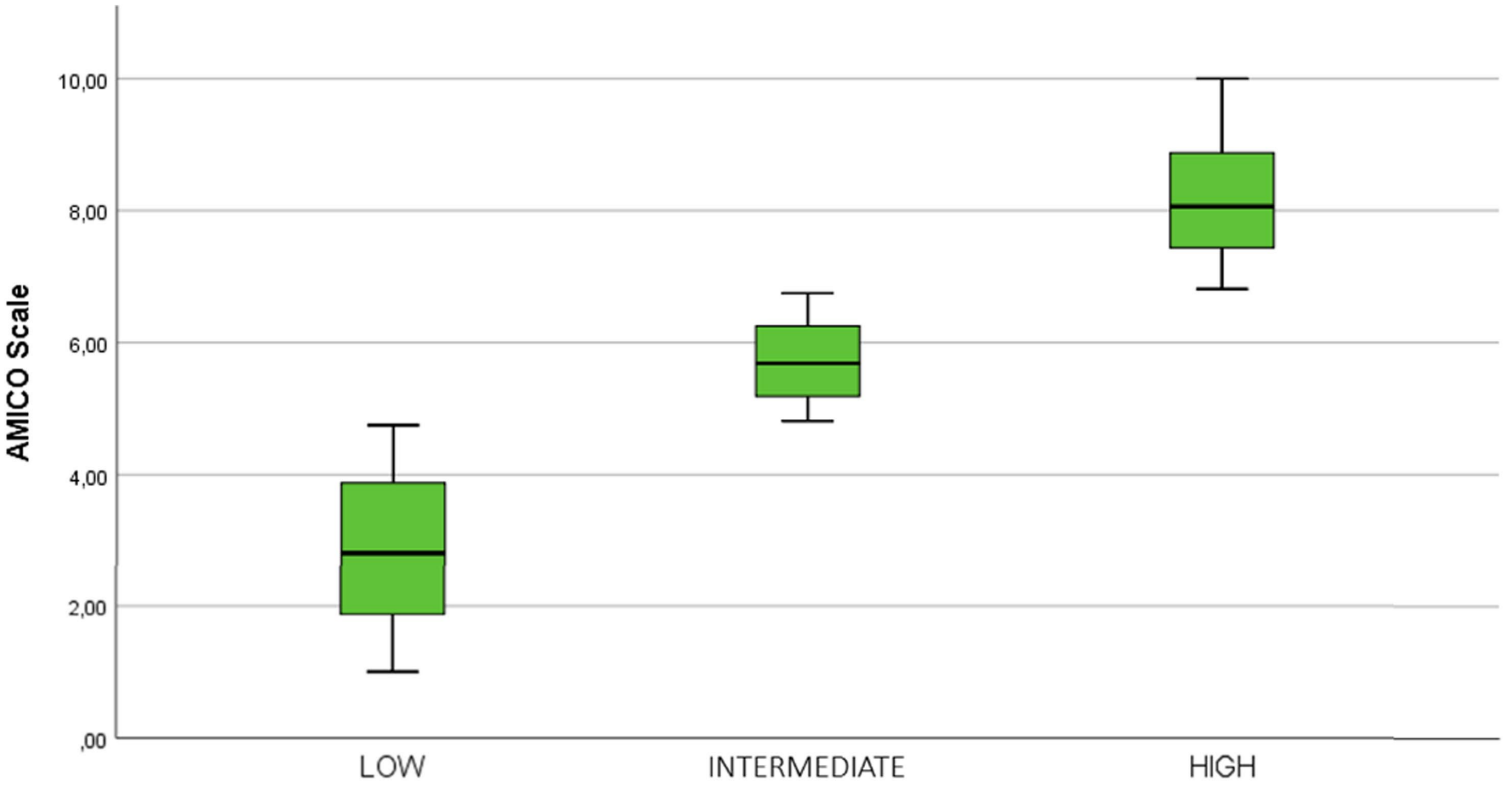
Distribution of the sample in levels of anxiety and fear of COVID-19.

On the other hand, the study of the percentiles and quartiles with respect to the distribution of the mean scale scores allowed the identification of three indicated levels of anxiety, using a box-and-whisker plot. A low level was identified, with scores from 0 to 4.7 points, an intermediate level with scores from 4.71 to 6.7 points, and a high level, from 6.71 to 10 points (Figure 2). The statistical significance of the variances between each pair of analyzed levels always gave a value of *p* = 0.000, confirmed with the Mann–Whitney U statistic; therefore, it can be confirmed that there are significant differences between the levels identified and their relevance.

## 4. Discussion

The aim of the present study was the construct validation of the AMICO_UK scale in the United Kingdom in order to assess the presence of fear and anxiety of COVID-19 in this population group.

The outcomes obtained have provided optimal results in the goodness-of-fit indices for the construct validation of the scale and its overall corrected reliability, and the goodness-of-fit and reliability values were very similar to those obtained in the initial study in Spain (20). Likewise, the present study also provides more refined reliability data, as it considers new calculations using McDonald’s omega (34), and therefore more robust reliability data.

Furthermore, although the items have high weights in both factors, the CFA considering a single factor has been shown to have a worse model fit than the proposed two-factor factor structure.

In addition, the unidimensionality of the scale has been demonstrated by means of a bifactor analysis, which justifies the obtaining of a single final scale score.

In relation to scales measuring fear of COVID-19, the so-called “Fear of COVID-19 Scale” (FCV-19) (14), widely validated in several countries since its creation, was also validated in the United Kingdom and New Zealand population (36).

However, this scale only measures the ‘fear’ construct, unlike the AMICO_UK scale, which also measures anxiety (20). This is the added value of the scale, as it provides information about two different but related constructs that could condition adherence behaviours to safety recommendations issued by governments in terms of public health.

The results of the bivariate analysis may suggest that the impact of the COVID-19 pandemic on mental health could be studied from a social perspective. In this regard, a study from University College London, which started after the COVID-19 pandemic, also concluded that women, young people, socially disadvantaged groups, and people with pre-existing mental health problems have been the most affected by the pandemic in terms of mental health, and confirms these outcomes (37). Similarly, another study on the United Kingdom population also found that young people, women, people with children at home, people with pre-existing mental health problems, and those with low economic status showed high levels of depression and anxiety at the onset of the confinement (21).

The fact that women showed higher anxiety rates than men was also confirmed in the present study with the AMICO scale scores. This could be due to the fact that most of the household care tasks fall on women; this was exacerbated by the alarm and lockdown situation and the consequent school closures, and perhaps women have experienced an increase in the number of tasks (20). Similarly, according to the results of the study by Pierce et al. in the specific case of the United Kingdom, women had higher rates of anxiety than men during the pandemic (11). Another study, albeit on a sample of people from the United States, also concludes that women and non-binary people, as well as people with pre- existing physical and mental health conditions, had higher levels of depression and anxiety after the COVID-19 pandemic was declared (38).

On the other hand, for the economically well-off, the cessation of commuting, changes in education and work activities, and increased time with family may have reduced stress and improved mental health and well-being (39). However, for the more economically vulnerable part of the population, the worsening employment situation might have worsened the mental distress that already existed in this group (11).

Although in the present study age was not a determining factor, it is true that, in general, in the articles reviewed, the rates of mental distress were worse for young people; this was the case in a study that highlights that younger populations tend to have, in general, worse mental health outcomes (40). One explanation for this phenomenon could be that many young students experienced worse academic performance and significant changes in their daily routine during a pandemic (41). In the United Kingdom, anxiety and depression symptomatology has been reported to be highest in young adults (<35 years), improving progressively with increasing age, with the lowest levels found in people aged 60 years and older (42). Similarly, another longitudinal study of United Kingdom households including participants from England, Wales, Scotland, and Northern Ireland, which included pre- pandemic data, also showed that the mental health of the population deteriorated in the early stages of the pandemic, pointing to higher rates of anxiety, depression, stress, suicide risk, and post-traumatic stress disorder (11). Rates of suicidal ideation also increased during the first weeks of confinement, 11% higher than in the previous year.

Weekly rates of suicidal ideation were also higher in the United Kingdom than elsewhere (43).

What is surprising, however, is that rates of self-harm ideation increased at the same time as COVID-19 restrictions decreased (44). This may translate into delayed true effects of the pandemic. However, while other studies have shown an overall increase in mental distress in people aged 16+ in the United Kingdom compared to the previous year (11), it is very difficult to estimate what the long-term mental health effects will be more than 2 years into the pandemic. However, we do have data from other epidemics such as SARS in 2003 (12).

For example, a study in Hong Kong, one of the regions of China most affected by SARS, found that there was a 30% increase in suicide in people aged 65 years and older, that about 50% of those who recovered from SARS remained anxious, and that 29% of health care workers experienced emotional distress. In addition, people who overcame this illness were at risk of post-traumatic stress disorder and depression (45). It is worth noting that the global impact of COVID-19 cannot be compared to that of SARS, and it can be concluded that the data on mental health effects will be much more significant.

For all these reasons, there is a need to continue researching the long-term consequences of COVID-19 on a daily basis in order to be able to offer the population the necessary resources at all times to mitigate the damage at a mental level. In this regard, the present study proposes a scale that measures the constructs of anxiety and fear of COVID-19 unidimensionally, which may be beneficial not only for measuring the prevalence of anxiety and fear of COVID-19 in the United Kingdom, but it can also be used in the experimental study to capture changes in anxiety and fear of COVID-19 that are related to adverse mental health outcomes.

As for the limitations of this study, it is worth highlighting the non-probabilistic sampling through which the individuals in the sample were selected as it may affect the generalisability of the data. Furthermore, since the data collection tool was telematic, there may also be an accessibility bias, as older people and/or those at risk of social exclusion may not have been able to answer the questionnaire. On the other hand, the greater number of women among the sample selected for the study. Therefore, it is necessary to examine, through a new field study, whether the variable “sex” may possibly have such an effect on the validation of the construct of the AMICO_UK scale from a gender perspective. On the other hand, further studies are needed to determine the criterion validity of the scale by calculating ROC curves, and which may also allow for predictive analysis and other regression measures. This is therefore another limitation of the present study.

However, in relation to the impact of the present study and its application to the practice, the validation of this scale could be an appropriate tool to measure the mental health impact that COVID-19 will have on the United Kingdom population in the long term, following the natural evolution of the pandemic. It is therefore positioned as a tool that could allow analysing this long-term impact, as there still are no prospective studies on this issue.

## 5. Conclusion

The AMICO_UK scale has adequate construct validity as an instrument to measure the presence of anxiety and fear related to COVID-19 in the United Kingdom population.

The majority of the United Kingdom population had low levels of anxiety and fear at the time the scale was administered. Due to the strong relationship between the impact of the COVID-19 pandemic and the social characteristics of the population, there is a need to assess specific population groups with the AMICO_UK scale with the aim of providing more targeted programmes and helping to improve and restore the mental health of the United Kingdom population.

## Data availability statement

The raw data supporting the conclusions of this article will be made available by the authors, without undue reservation.

## Ethics statement

The studies involving human participants were reviewed and approved by Permission to conduct this study was obtained from the National Health Service (NHS) Committee, Ref. 20/HRA/369. The patients/participants provided their written informed consent to participate in this study.

## Author contributions

CM-T, RA-C, JF-R, YN-A, JC-R, and JG-S: conceptualization. CM-T, JC-R, and RA-C: data curation. CM-T, RA-C, JG-S, JF-R, JC-R, and YN-A: formal analysis. CM-T, RA-C, JG-S, JC-R, and JF-R: investigation. CM-T, JG-S, RA-C, JF-R, and YN-A: methodology. JG-S and CM-T: project administration. RA-C, JG-S, JC-R, and YN-A: resources. CM-T, RA-C, JG-S, and YN-A: software. JG-S, RA-C, JF-R, JC-R, and YN-A: supervision. RA-C, JG-S, and JF-R: validation. JF-R and JC-R: visualization. RA-C, CM-T, JC-R, and JG-S: writing—original draft. RA-C, JG-S, JF-R, and YN-A: writing—review and editing. All authors contributed to the article and approved the submitted version.

## Conflict of interest

The authors declare that the research was conducted in the absence of any commercial or financial relationships that could be construed as a potential conflict of interest.

## Publisher’s note

All claims expressed in this article are solely those of the authors and do not necessarily represent those of their affiliated organizations, or those of the publisher, the editors and the reviewers. Any product that may be evaluated in this article, or claim that may be made by its manufacturer, is not guaranteed or endorsed by the publisher.
